# Association of Frontline Worker-Provided Services with Change in Block-Level Complementary Feeding Indicators: An Ecological Analysis from Bihar, India

**DOI:** 10.1371/journal.pone.0166511

**Published:** 2016-11-10

**Authors:** Aritra Das, Sanchita Mahapatra, Guntur Sai Mala, Indrajit Chaudhuri, Tanmay Mahapatra

**Affiliations:** 1 CARE India Solutions for Sustainable Development, H No. 14, Patliputra Colony, Patna, Bihar, PIN Code— 800013, India; 2 Department of Epidemiology, Fielding School of Public Health, University of California Los Angeles, 650 Charles Young Drive, South, Box 951772, Los Angeles, California, 90095, United States of America; TNO, NETHERLANDS

## Abstract

**Background:**

Insufficiencies in complementary feeding put infants and young children at increased risk of undernutrition. Till now, most Indian studies have looked at the individual level determinants of complementary feeding practices. We aimed to evaluate the association of frontline worker (FLW) provided nutritional counselling services, with change in community level indicators of complementary feeding practices among 9–11 month old children over time.

**Methods:**

The study data was obtained from five rounds of ‘Lot Quality Assurance Sampling’ survey in eight districts of Bihar, an impoverished Indian state. The surveys were conducted as evaluation exercises for the ‘Integrated Family Health Initiative (IFHI)’–a multi-faceted program aimed at improving the maternal and child health outcomes in Bihar. The main outcome indicators were—current breastfeeding, age-appropriate minimum frequency of semi-solid food, age-appropriate minimum quantity of semi-solid food, initiation of complementary feeding at the right age, and dietary diversity. Repeated measures analysis was performed to determine the association of changes in the outcome indicators with coverage of FLW-provided counselling services.

**Results:**

Visits by FLW, advices on age-appropriate frequency and handwashing were significant predictors of receiving age-appropriate frequency of feeding. The determinants of receiving age-appropriate quantity were—advices on age appropriate frequency and advices on handwashing. Receiving food support from AWC and FLW visits were significantly associated with initiating complementary feeding at the right age.

**Conclusions:**

The present study identified the critical elements among the different types of FLW-provided services. The study findings, from an economically and socially underdeveloped region of India, would inform the relevant programs about the nutritional counselling services that need to be emphasized upon for reducing the burden of childhood malnutrition.

## Introduction

According to the World Health Organization (WHO), complementary feeding is the process that takes place during the transition period following the recommended age of exclusive breast feeding (6 months) and prior to graduating to regular family food (~18 to 24 months).[1] It has been stressed in the literature that optimal nourishment during the infancy and early childhood is essential for ensuring that a child can develop to his/her full potential.[2, 3] Unless recommended otherwise, up to about six month age exclusive breastfeeding meets the criteria for providing ideal nutrition satisfactorily. Nevertheless, following this period, timely initiation of nutritionally-adequate, safe, age-appropriate complementary feeding is critical to meet the changing requirements of a growing child and his/her overall development.[4, 5]

Inadequacies in complementary feeding can put infants and young children at heightened risks of malnutrition, various infections and even mortality.[6] Besides other interventions targeted at malnutrition prevention and morbidity reduction among 6 to 24 months old children, strategies aimed at improving the coverage and quality of complementary feeding can go a long way in decreasing the burden of growth disorders e.g. stunting and helping overall nutritional status and development.[7] Ensuring optimal complementary feeding has been suggested as an essential step towards attaining the Sustainable Development Goal (SDG) regarding prevention of childhood malnutrition and, in turn, under-five mortality.[8,9] Predictors of inappropriate and inadequate complementary feeding practices have been reported from both the resource rich and poor countries.[10] Family (maternal) level determinants of complementary feeding include maternal age, marital status, education, occupation, available perinatal health care, health awareness, socio-economic status and area of residence; whereas some widely reported child level predictors were weight at birth, delivery type, birth order, and the use of pacifiers.[10–13]

Among the under-five children in India, which constitute approximately 10% of its total population, 43% are underweight and 48% suffer from stunting due to chronic undernutrition.[14] In fact, it is estimated that India accounts for every three out of ten stunted children in the World.[15] Amid such a grave scenario, there has been a concerted effort on the part of various governmental and collaborating agencies to improve the situation. A major step taken in this direction has been to equip the frontline workers (FLW)–a group of village level community health workers comprising of Anganwadi workers (AWW) and Accredited Social Health Activist (ASHA)–with appropriate knowledge and skills to counsel and support the mothers/caregivers about various maternal and child health issues including excusive breast feeding and complementary feeding.[16] These FLWs, working under India’s Integrated Child Development Services (ICDS) and National Health Mission (NHM) programs, reach rural women and families with various interventions through a combination of facility-based, outreach and community-based contacts. Besides the over-arching goal of improving the maternal and child health indicators, an important long-term objective of this endeavor is to help Indian children meet the WHO Child Growth Standards.[17]

Prior studies conducted in India have reported that an association exists between infant and young child feeding practices and childhood undernutrition.[18, 19] However, Bhandari et al reported that an intervention to improve the complementary feeding practices yielded very limited benefit in terms of physical growth among 6 to 18 month old children.[20] It should be noted that, thus far, the Indian studies on this topic had mostly focused on individual level determinants of complementary feeding practices. The impact of various childhood nutritional interventions offered under NHM and ICDS programs are yet to be thoroughly explored. The predicted pattern of changes in the complementary feeding indicators at the community level with changes in coverage of nutrition interventions remains to be investigated. We understand that, as the interventions are already in place across most parts of the country, it would not be possible to assess their efficacy using a community based trial. However, as reach of these services differ in different states of India and even within states, a viable alternative would be to evaluate the impact on complementary feeding indicators with time in the regions with varying coverage. To meet this objective, the present study analyzed sub-district level data, collected under the ‘Integrated Family Health Initiative (IFHI)’ program in the state of Bihar, to evaluate the association between coverage of FLW-administered nutritional counselling and complementary feeding indicators.

## Materials and Methods

### Ethical approval

The current study was approved by the ‘Institutional Committee for Ethics and Review of Research’ of Indian Institute of Health Management Research (www.iihmr.org), Jaipur, India. Informed consent (including signature or left thumb impression of the respondent) was collected from each agreeing participant before the interview and measurements, after explaining the details of the study in a language that they could understand.

### Study sample

The current analysis used data from five rounds of the ‘Lot Quality Assurance Sampling (LQAS)’ survey conducted as part of the IFHI program in Bihar.[21] LQAS is a small sample survey design based on binomial distribution.[22–24] This sampling design was primarily conceived as a quality assurance measure in the industrial setting to identify the batches of products (or lots) with an unacceptable number of defective items coming off a production line.[25, 26] The design was later adapted to evaluate public health interventions and services, especially in developing countries.[27, 28] Prior studies suggest that LQAS is an efficient design to identify the program areas or communities having inadequate service coverage, at a fraction of the cost of traditional surveys.[28–31] Therefore, in the context of Bihar, one of the poorest states in India, LQAS was considered a suitable design for evaluation of the IFHI program.

The five rounds of LQAS surveys were carried out between December 2011 and December 2013 in 137 blocks of eight randomly selected districts (out of total 38 districts) in Bihar. The sampling ‘lots’ in the surveys were the blocks or sub-districts, which were the unit of programmatic intervention. The survey periods and district-wise distribution of blocks and respondents are presented as supporting information (S1 Table). A multi-stage sampling methodology was used during the process. The sampling methodology has been described previously.[32] In short, from each of the 137 blocks 19 Anganwadi Center (AWC)–village level institutions providing basic health care services—areas were selected randomly through probability proportional to size (PPS) sampling. From each selected AWC area, four eligible households were identified through systematic random sampling. An eligible household was defined as that containing mothers with babies aged 0–23 (0–2, 3–5, 6–8, and 9–11 months) completed months. Using this strategy, for each survey round, altogether 2603 (137×19) AWCs were selected from the 8 study districts and from each of these AWCs a mother belonging to each of the four age sub-groups, as per age of the child, was interviewed. We used the dataset for mothers with 9 to 11 months old child for the present analysis.

### Outcome measures—complementary feeding indicators

The estimated block-wise performance (based on sampled population) on five complementary feeding indicators among 9 to 11 month old children—obtained from each round of LQAS—were used as outcome measures in our analysis. These outcome indicators were– 1) current breastfeeding, 2) receiving age-appropriate minimum frequency (≥3 times/day) of semi-solid food, 3) receiving age-appropriate minimum quantity (≥300ml/day) of semi-solid food, 4) initiation of complementary feeding at the right age (6–8 months), and 5) receiving dietary diverse complementary feeding. Only cereal-based semi-solid food fed to the child in a separate bowl by an adult was considered as complementary food. For age of initiation of complementary feeding, we only considered the responses from those mothers who started giving semi-solid food from 6^th^ month onwards. If anyone initiated complementary feeding earlier, for any reason, their responses were not accounted for. Dietary diversity was defined as receiving foods from at least four different food groups as part of complementary feeding during the previous week.[33] The block-wise proportions of above outcome indicators were converted to percentages and treated as continuous variables.

### Intervention measures

We used the estimated block-wise coverage (in percent) of various interventions, which were targeted at infant feeding practices and delivered through FLWs, as the predictor variables in our analysis. These interventions were– 1) receiving food support from the local AWC, 2) being visited by an FLW during the previous month, 3) ever receiving advice on age-appropriate frequency of complementary feeding (at/after 6 months age) from FLW, 4) ever receiving advice on age-appropriate quantity of complementary feeding (at/after 6 months age) from FLW, 5) ever receiving advice from FLW on washing hands prior to feeding/preparing food for the child. Besides AWW and ASHA workers, FLWs in the study area also comprised of Auxiliary Nurse Midwives (ANM). An FLW visit was defined as a home visit when an FLW interacted with the responding mother (interactions with other family members not counted) and during which some health-related discussion took place. The estimates of block-wise coverage of the above interventions were obtained from the last iteration of LQAS survey i.e. fifth round.

### Covariate measures

These measures were block level parameters of caste, religion, wealth (asset index), house type & parent's education. For caste, we estimated the percentage of population in each block that belonged to the marginalized castes [scheduled castes (SC) / scheduled tribes (ST) / other backward castes (OBC)]. In case of religion, percentage of Hindu population in each block was used. As for educational status of parents, we estimated the percentage of fathers and mothers in each block who received school education above eighth standard. We further estimated the block-wise proportion of population living in a ‘pucca’ house (house entirely built from bricks). We used an asset index (AI) as surrogate of economic status of study respondents. In order to create this index, we first inquired about possession of 25 different household assets. A relative weight was then assigned to each of these assets and an aggregated score was calculated for each household by adding the weighted score for each asset possessed by the household. The cumulative asset scores thus obtained were then log-transformed to create AI. At the block level we created a variable that captured the percentage of households in each block that belonged to the highest tertile of AI. Similar to intervention measures, the data for covariate measures were obtained from the fifth round of LQAS survey.

### Statistical analyses

Descriptive analysis was carried out to determine the distribution of socio-demographic and complementary feeding related characteristics in the study blocks. For description of block level characteristics, we dichotomized the blocks into the ones with ≥75% prevalence of a particular characteristic and those with <75% prevalence of the same characteristic. As there were no blocks in which ≥75% population belonged to the highest tertile of AI or where ≥75% reported receiving advice on age-appropriate quantity of complementary feeding, for these two characteristics we reduced the cut-off to ≥50%.

In order to determine the associations of block level outcome indicators with different interventions, we fitted separate longitudinal models for every combination of outcomes and interventions. The unadjusted models had specific outcome indicator as the dependent variable and a particular intervention as the sole independent variable (fixed effect), whereas the adjusted models also included caste, religion, asset index and mother’s education as independent variables. In addition, models were also adjusted for survey rounds. Except survey rounds, all other variables in the models were treated as continuous measures. These repeated measures model were fitted using *SAS Proc Mixed* with ‘Maximum Likelihood Estimation’ method. We employed ‘Compound Symmetry’ covariance structure for fitting the models whereby we assumed that the variance of the outcome indicator in question was same across all measurements at the block level and that the correlation between any pair of measurements (between any two survey rounds) was the same. The analysis generally followed that described in Weiss (2005).[34]

## Results

Of the 137 surveyed blocks, Hindus accounted for 75% or more population in 113 (83%) blocks. In 126 (92%) blocks 75% or greater proportion of the total population belonged to the marginalized castes (SC/ST/OBC), and there were 92 (67%) blocks where ≥75% residents lived in a ‘pucca’ house. The block level socio-demographic characteristics and parameters related to FLW-provided services are depicted in Table 1.

**Table 1:** Categorization of blocks in the state of Bihar as per socio-demographic characteristics and complementary feeding related service coverage.Bihar, 2011–13 (N = 137)^1^.

Characteristic	Frequency	Percent (%)	95% CI of predicted %
Blocks where Hindus accounted for ≥75% population	113	82.48	76.04, 88.93
Blocks where people of marginalized castes (SC/ST/OBC) accounted for ≥75% population	126	91.97	87.36, 96.57
Blocks where ≥75% residents lived in 'Pucca' houses	92	67.15	59.19, 75.12
Blocks where ≥75% mothers of 9 to 11 month old children have not studied beyond 8th standard in school	105	76.64	69.46, 83.82
Blocks where ≥75% fathers of 9 to 11 month old children have not studied beyond 8th standard in school	49	35.77	27.64, 43.99
Blocks where ≥50% families belonged to highest tertile of asset index^2^	32	23.36	16.18, 30.53
Blocks where ≥75% mothers of 9 to 11 month old children reported that they wash their hands before feeding the child	108	78.83	71.91, 85.76
Blocks where ≥75% mothers of 9 to 11 month old children reported receiving food supplement from AWC	21	15.33	9.22, 21.44
Blocks where ≥75% mothers of 9 to 11 month old children reported having their child weighed during previous month	3	2.19	0.00, 4.67
Blocks where ≥75% mothers of 9 to 11 month old children reported being visited by (FLW) ASHA/AWW/ANM during the previous month	42	30.66	22.84, 38.47
Blocks where ≥75% mothers of 9 to 11 month old children reported receiving advice on age-appropriate frequency of complementary feeding by FLW	7	5.11	1.37, 8.84
Blocks where ≥50% mothers of 9 to 11 month old children reported receiving advice on age-appropriate quantity of complementary feeding by FLW^3^	3	2.19	0.00, 4.67
Blocks where ≥75% mothers of 9 to 11 month old children reported receiving advice on handwashing before feeding the child from FLW	20	14.59	8.61, 20.59

^1^Based on responses of mothers of 9 to 11 month old children.

^2^There were no blocks where ≥75% belonged to highest tertile of the asset index.

^3^There were no blocks where ≥75% reported receiving advice on age-appropriate quantity of complementary feeding.

**Fig 1:**
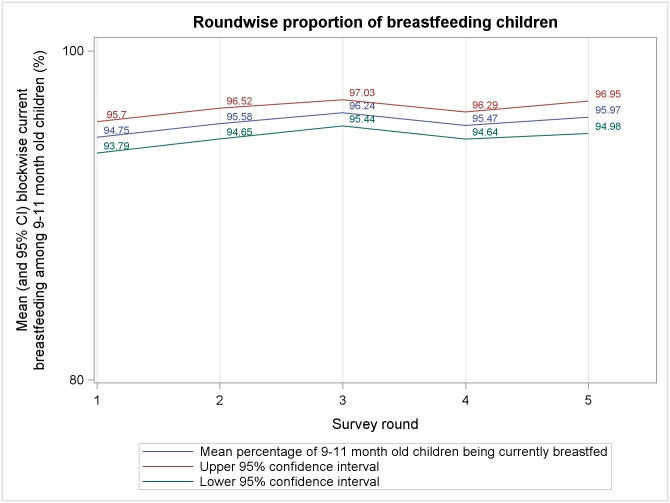
Mean block level percentages of 9–11 months old children being currently breastfed.

**Fig 2:**
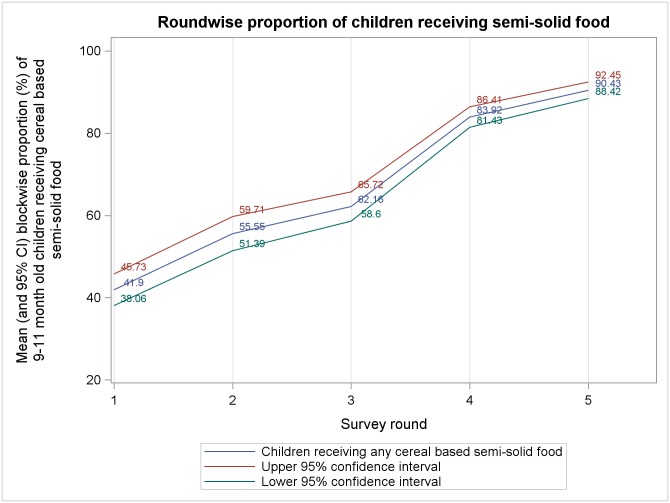
Mean block level percentages of 9–11 months old children receiving semisolid food.

**Fig 3:**
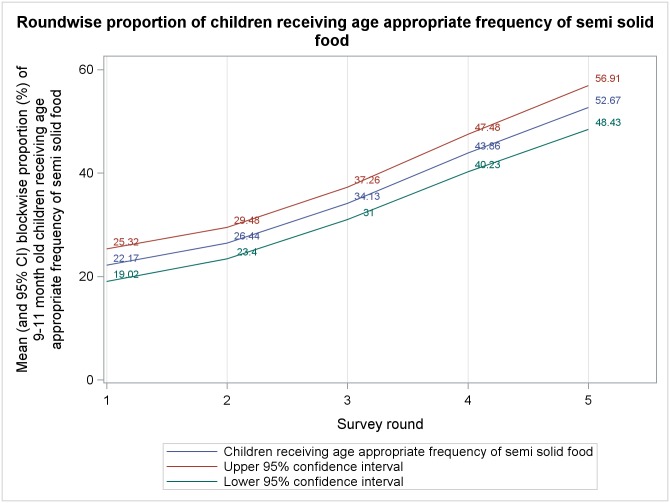
Mean block level percentages of 9–11 months old children receiving age-appropriate frequency of semisolid food.

**Fig 4:**
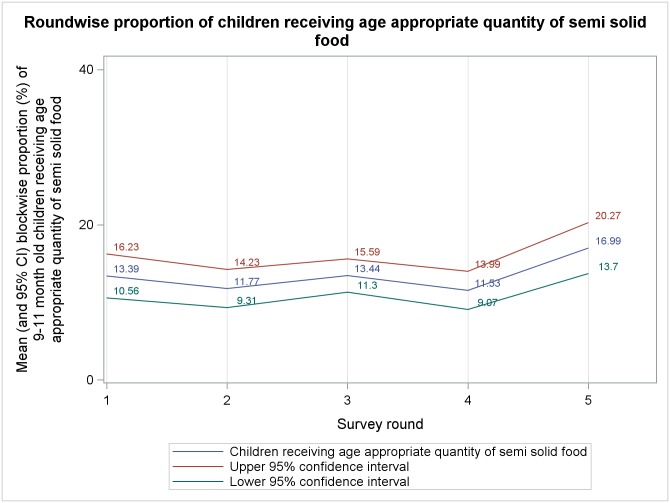
Mean block level percentages of 9–11 months old children receiving age-appropriate quantity of semisolid food.

**Fig 5:**
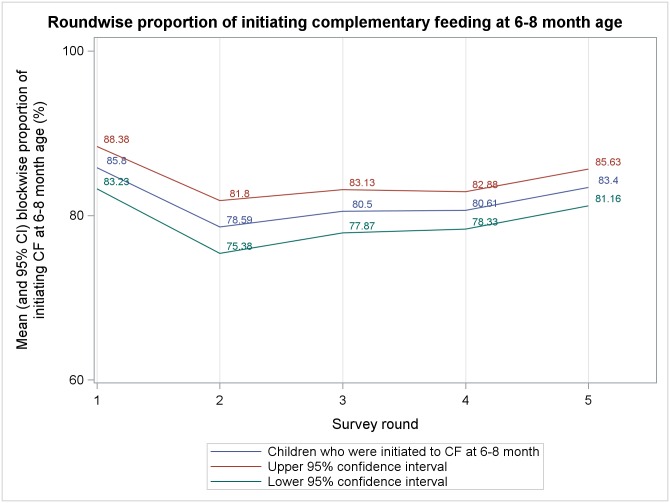
Mean block level percentages of 9–11 months old children who were initiated to complementary feeding at the age of 6–8 months.

**Fig 6:**
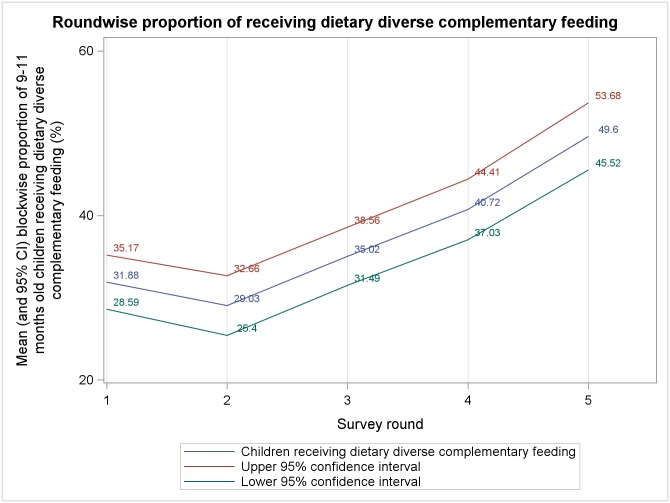
Mean block level percentages of 9–11 months old children receiving dietary diverse complementary feeding.

Mean block level percentages, from five rounds of survey, of different complementary feeding indicators among 9–11 months old children are presented in Figs 1–6. A significant upward trend, over time, was observed for–receiving any semi-solid cereal-based food (*p* <0.01), receiving age appropriate frequency of semi solid food (*p* <0.01) and receiving diverse diet (*p* <0.01).

Crude (unadjusted) and adjusted parameter estimates (β) and 95% confidence interval (CI) from repeated measures analyses on predictors of change in complementary feeding indicators among 9 to 11 months old children across the survey rounds are presented in Table 2. In the unadjusted analysis, we found that receiving advice on age appropriate frequency of complementary feeding showed significant positive association with current breastfeeding (β: 0.02, 95% CI: 0.00, 0.04). In the domain of age appropriate frequency of complementary feeding, it was revealed that the proportion of mothers visited by FLW during the previous month (β: 0.08, 95% CI: 0.00, 0.15), receiving advice on age-appropriate frequency of complementary feeding from FLW (β: 0.19, 95% CI: 0.09), and receiving handwashing advice from FLW (β: 0.13, 95% CI: 0.04, 0.21) were significant determinants. Further, FLW visits during the previous month (β: 0.05, 95% CI: 0.00, 0.11), receiving advices from FLW on age-appropriate frequency of complementary feeding (β: 0.11, 95% CI: 0.05) and advice on handwashing prior to feeding/preparing food (β: 0.08, 95% CI: 0.02, 0.13) were revealed as significant positive predictors of receiving age-appropriate quantity of complementary feeding. Initiating complementary feeding at the recommended age (6–8 month) was found to be positively associated with receiving food support from AWC (β: 0.11, 95% CI: 0.03, 0.18) and FLW visit during the previous month (β: 0.10, 95% CI: 0.05, 0.15).

**Table 2:** Crude and adjusted associations of interventions with change in block level indicators related to complementary feeding among 9 to 11 months old children across survey rounds. Bihar, 2012–13 (N = 137).

Interventions (block level coverage)	Current breastfeeding		Receiving age appropriate minimum frequency of complementary feeding		Receiving age appropriate minimum quantity of complementary feeding		Initiating complementary feeding at 6–8 month age		Receiving dietary diverse complementary feeding	
	β (95% CI)		β (95% CI)		β (95% CI)		β (95% CI)		β (95% CI)	
	Unadjusted	Adjusted^#^	Unadjusted	Adjusted^#^	Unadjusted	Adjusted^#^	Unadjusted	Adjusted^#^	Unadjusted	Adjusted^#^
Receiving food support from AWC	0.00 (-0.02, 0.03)	0.01 (-0.01, 0.03)	0.07 (-0.04, 0.20)	0.06 (-0.06, 0.18)	0.07 (-0.01, 0.16)	0.07 (-0.02, 0.15)	0.11* (0.03, 0.18)	0.10* (0.02, 0.18)	0.06 (-0.07, 0.20)	0.02 (-0.11, 0.16)
Visit by FLW during previous month	0.01 (-0.01, 0.02)	0.01 (0.00, 0.03)	0.08* (0.00, 0.15)	0.09* (0.01, 0.17)	0.05* (0.00, 0.11)	0.05 (-0.01, 0.11)	0.10* (0.05, 0.15)	0.09* (0.04, 0.14)	0.04 (-0.05, 0.13)	0.03 (-0.06, 0.12)
Receiving advice on age appropriate frequency of complementary feeding from FLW	0.02* (0.00, 0.04)	0.02* (0.00, 0.04)	0.19* (0.09, 0.28)	0.18* (0.09, 0.27)	0.11* (0.05, 0.17)	0.11* (0.04, 0.17)	0.06 (-0.03, 0.15)	0.05 (-0.02, 0.12)	0.05 (-0.07, 0.17)	0.05 (-0.06, 0.16)
Receiving advice on age appropriate quantity of complementary feeding from FLW	0.03 (-0.01, 0.07)	0.02 (-0.01, 0.06)	-0.05 (-0.24, 0.14)	-0.02 (-0.20, 0.17)	-0.01 (-0.14, 0.12)	-0.03 (-0.16, 0.10)	-0.01 (-0.19, 0.17)	-0.02 (-0.16, 0.11)	-0.09 (-0.30, 0.14)	-0.08 (-0.29, 0.12)
Receiving handwashing advice from FLW	0.01 (0.00, 0.03)	0.02* (0.00, 0.03)	0.13* (0.04, 0.21)	0.14* (0.05, 0.22)	0.08* (0.02, 0.13)	0.08* (0.02, 0.14)	0.03 (-0.05, 0.11)	0.05 (-0.01, 0.11)	0.07 (-0.03, 0.16)	0.08 (-0.02, 0.17)

β = Parameter estimate from repeated measures regression analysis

^#^Adjusted for survey rounds and block level parameters of caste, religion, wealth (asset index) & mother's education.

Positive parameter estimates indicate that block level performance of an indicator is positively associated with increasing coverage of interventions (and vice versa).

*Statistically significant (p≤0.05). The point estimates and 95% CI were rounded to 2 decimals places.

Results from adjusted analysis revealed that being advised by FLWs on age-appropriate frequency of complementary feeding was a significant positive predictor of current breastfeeding (β: 0.02, 95% CI: 0, 0.04). Moreover, receiving advice from FLW on handwashing, which was not significant in the crude analysis, emerged as a significant predictor of increasing proportion of breastfeeding (β: 0.02, 95% CI: 0, 0.03). FLW visit in the previous month (β: 0.09, 95% CI: 0.01, 0.17), receiving advice on age-appropriate frequency (β: 0.18, 95% CI: 0.09, 0.27), and receiving handwashing advice from FLW (β: 0.14, 95% CI: 0.05, 0.22) were significantly associated with block level proportions of age-appropriate frequency. For age-appropriate quantity of complementary feeding, advices from FLW on age appropriate frequency (β: 0.11, 95% CI: 0.04, 0.17) and advices on handwashing (β: 0.08, 95% CI: 0.02, 0.14) emerged as significant positive determinants. Advice from FLW on age appropriate quantity of complementary feeding was found to have no significant association with either age-appropriate quantity or frequency, in both the unadjusted and adjusted models. Further, in the domain of initiation age for complementary feeding, receiving food support from AWC (β: 0.10, 95% CI: 0.02, 0.18) and FLW visit during the previous month (β: 0.09, 95% CI: 0.04, 0.14) were significantly associated with initiating at the right age (6–8 months). None of the FLW-provided services were significantly associated with changes in block level proportions of children receiving dietary diverse complementary feeding, neither in crude nor in adjusted analysis.

## Discussion

The present study was conducted as part of an evaluation exercise for the IFHI program, which sought to improve the maternal and child health outcomes in Bihar, an economically backward state. An important goal of the ‘outreach’ component of the IFHI program was improvement in frontline service delivery. Through an integrated package, the program worked in close collaboration with the Dept. of Health (DoH) and the Social Welfare Dept. (SWD) to define specific tasks for different cadres of frontline workers—ASHA, AWW and ANM—and identified opportunities to integrate service delivery and reinforce critical messages and services. The nutrition-specific outreach interventions in IFHI included promotion of optimal maternal and children nutrition by delivering consistent behavior change messages from ASHAs during the birth planning sessions, AWWs at home visits or mothers’ groups, and ANMs at the antenatal care sessions or at ‘Village Health Nutrition Days (VHNDs)’. From the perspective of service delivery, the program developed quality standards for each cadre of provider, and adapted training materials, job aids and supervisory tools to emphasize context-specific packages of care.

In order to inform the FLW service delivery integration component of the IFHI program, the current analysis assessed the ecological associations between coverage of various FLW-provided, nutrition-related services (offered under NRHM/ICDS) and community level indicators of complementary feeding. The analysis used data from multiple time points employed repeated measures analysis technique to evaluate whether coverage of FLW-provided services predicted temporal changes complementary feeding practices. Although the immediate objective of this exercise was to inform the IFHI program, the overarching goal of our findings would be to bolster India’s efforts to meet its assigned SDG target pertaining to child survival and the prevention of malnutrition.

From analysis of trends in outcome indicators, we observed that the proportion of 9–11 month old children being currently breastfed remained very high (~95%) during all rounds of survey, albeit without any significant change across rounds. Therefore, our findings reiterate that in rural India breastfeeding continues to be almost universal.[35, 36] While breastfeeding scenario was heartening, we found that much lesser proportion of children were receiving minimum age-appropriate frequency and quantity of complementary feeding. Moreover, proportion of children receiving age-appropriate frequency remained higher than the proportion receiving age-appropriate quantity during every round of survey. This is similar to the findings of National Family Health Survey-3 (NFHS-3) among 6–11 month old children.[33] Also, there was a significant upward trend in receiving age-appropriate frequency of complementary feeding but same did not occur with age-appropriate quantity. We hypothesize that, owing to economic constraints, many families found it difficult to provide for appropriate quantity of semi-solid food to their children. However, even if the families were providing less than adequate quantity some of them divided the meal in smaller portions over the day to maintain frequency. Around 75%-85% children, in different rounds, who were exclusively breastfed were reportedly initiated to complementary food at right age (6–8 months). This was much higher than that reported in NFHS-3.[33] The higher proportion could be an artifact of recall bias as mothers of 9–11 month old children, many with little formal education, were inquired about the month of initiation of complementary feeding. Proportion of children receiving minimally dietary diverse meals (~29% to ~50% in different rounds) were also higher than that previously reported[33] and it also showed an increasing trend over time. This could be due to the fact that with increasing awareness level about complementary feeding, owing to interventions taken under ICDS and NRHM, some inexpensive locally grown fruits and vegetables got their places into the meals of infant and children. Such an action might have increased the diversity in infant feeding which previously leant heavily towards more expensive milk, milk-based items and infant formulae because of social customs.[37, 38]

Among the interventions tested for, we found that increasing coverage of food support from AWC was positively associated with block level indicator for initiation of complementary feeding at the right age. However, the food support was not associated with either frequency or quantity of complementary feeding. We assume that receiving additional food supply might initially promote provision of additional food for the infant, but such support might be too insufficient to sustain practices of adequate age-appropriate complementary feeding, especially among the poorer section of the society. Also, the quality of food offered through India’s public distribution system have been subjected to numerous criticisms over the years.[39] Even if food support was received, in case it was not up to the mark, it might not have translated to actual feeding of the child.

Increasing block level coverage of home visits by FLW, during which health-related discussions took place, was positively associated with the proportion of families in which children received age-appropriate frequency of meals and were initiated to complementary feeding at the right age. Moreover, proportion of children being breastfed also showed positive association and was very close to reaching the level of significance. Visits during which complementary feeding related discussions took place might have had a direct effect on the above outcome indicators. Besides, even if an FLW visit did not involve any discussion on complementary feeding, any other discussions related to child health likely to have caused the mothers seek health information from local health facilities and, in turn, indirectly affected the above performance indicators.

Curiously, while block level exposure to advice on age-appropriate frequency of feeding was positively associated with all complementary feeding indicators, including significant associations with breastfeeding, frequency and quantity of complementary feeding, advice on age-appropriate quantity of feeding did not have any significant positive effects on any of the study outcomes. Our understanding is that advice on frequency is much easier to disburse and to understand by the audience, compared to advice on appropriate quantity. While the former involves informing the mother about distributing the daily feeding process into three or more servings, the latter demands careful measuring of food using common household utensils. Moreover, it is intuitive that the mothers, who followed the instructions on appropriate frequency and provided ≥3 meals/day, likely ended up providing the appropriate quantity of food, too. However, advice on appropriate quantity might not always translate to appropriate frequency. Therefore, from the program point of view, it is imperative that advice on age-appropriate frequency of feeding be emphasized upon to achieve desired performance related to complementary feeding. Also, the fact that block level penetration of receiving advice on age-appropriate quantity was quite low (in only 3 blocks ≥50% mothers reported receiving such advice) might have prevented it from being significantly associated with complementary feeding indicators in statistical analysis. Consequently, we further recommend that, during training of FLWs, a simplified approach of disbursing advice on appropriate quantity be taken up, which can be helpful in overcoming barriers related to such advice at the communicator and audience levels.

Interestingly, block level coverage of handwashing advices demonstrated a significant positive association with current breastfeeding, age-appropriate frequency and quantity of complementary feeding. We speculate that advices about washing hands, prior to feeding/preparing food for child, serve as surrogate for the entire group of interventions related to complementary feeding. The process of handwashing, which takes place before feeding the child, is a precursor to the indicators of complementary feeding under consideration. Therefore, mothers who followed the advice on handwashing were also somewhat likely to abide by other advices related to their child’s feeding. Furthermore, health seeking behavior might act as a confounder in the relation between advice on handwashing and complementary feeding indicators. Handwashing is often associated with better health seeking behavior [40] and the mothers with better health seeking behavior are more likely to be diligent about feeding practices for their child. Thus, we hypothesize that handwashing counselling could have a greater than anticipated effect on curbing childhood malnutrition—as it not only reduces risks of infection but also promotes breastfeeding and complementary feeding practices. However, this hypothesis needs to be corroborated through community-based trials having sufficient power.

In our analysis, crude or adjusted, none of the services offered through FLW showed a significant association with block level indicator for minimum dietary diversity in complementary food. It is not entirely unexpected as mother’s decision to add diverse food item in her child’s meal does not only depend on her knowledge and familial/social customs but also on the availability and/or affordability of the food items. Even the food support provided through local AWC might be sub-par and not diverse enough, which is especially true for Bihar.[41] Interventions that promote local control and autonomy over selection of food items along with awareness building may prove helpful in this regard.[42]

We acknowledge presence of several limitations in the current study. First, because of the serial cross-sectional nature of the analyzed data, inferences about causal associations might be hampered by lack of temporality. It was often unclear whether the interventions took place prior to the concerned outcome. Second, to assess the associations of block level coverage of interventions with changes in block level outcome indicators, ecologic analysis methods were adopted. As the study sample changed with each round of LQAS survey it was not possible to link individuals to block level parameters, and, hence, we could not adjust for confounders at individual level. Therefore, our results might suffer from ‘ecologic fallacy’. This also restricted us from making inferences about individual level associations between interventions and outcome measures. Third, the statistical power estimation for the LQAS survey was conducted assuming individual level analyses. Therefore, the data used for current analyses might have had insufficient power for block-level (ecological) analyses. This might explain the reason behind many positive associations in our analyses failing to achieve statistical significance. Finally, although the data for the study arose from a multi-stage sample survey, its representativeness may not hold for analysis at the block level. Therefore, we should practice caution about the generalizability of our study findings.

Despite the limitations, our study also had some important strengths. Data from multiple rounds of a large-scale survey allowed us to measure the change in several complementary feeding indicators over time. Data quality of the LQAS survey was assured through rigorous training and use of a uniform protocol at every site, which, we expect, helped to minimize variation across interviewers. Moreover, the current analysis used mixed modelling technique for data collected at multiple time points. This approach allowed us to assess predictors of change in outcome indicators while adjusting for common covariates.

Complementary feeding practices are essential components of the care received by infant/young children, irrespective of their socio-cultural, economic and demographic backgrounds, and form a key point of intervention against childhood malnutrition. Although the childhood malnutrition scenario in India improved substantially during the past few decades[43], achieving the nutrition-related SDG targets will require a stronger and sustained effort towards optimal breast feeding and complementary feeding practices.[44] The IFHI program through its integrated service delivery component aimed to improve the quality of various FLW-provided services—including nutritional counselling—in Bihar. The current study, in order to inform the IFHI and other relevant programs, identified the critical elements among a range of existing FLW-provided childhood feeding related services. A well-designed community-based trial may overcome the methodological limitations of the current study and further the understanding on the casual association between community level nutritional counselling and complementary feeding practices. Such an effort may additionally help to explore the efficacy of complementary feeding related interventions in improving malnutrition indicators.

## Supporting Information

S1 Table Periods of LQAS survey rounds and district-wise distribution of blocks and respondents. (DOCX)
