# Supplementary material for: Association of Frontline Worker-Provided Services with Change in Block-Level Complementary Feeding Indicators: An Ecological Analysis from Bihar, India
Source: PLoS One. 2016 Nov 10;11(11):e0166511. doi: 10.1371/journal.pone.0166511 (PMC5104399; doi:10.1371/journal.pone.0166511)
Supplement: S1 Table — (DOCX) [file pone.0166511.s001.docx]

**S1 table. Periods of LQAS survey rounds and district-wise distribution of blocks and respondents.**

| **District** | **No. of Blocks** | **LQAS Rounds and time periods** | | | | |
| --- | --- | --- | --- | --- | --- | --- |
|  |  | **R1 (Dec'11 - Feb'12)** | **R2 (Oct'12 - Dec'12)** | **R3 (Feb'13 - May'13)** | **R4 (Jun'13 - Aug'13)** | **R5 (Oct'13 - Dec'13)** |
| Patna | 23 | 437 | 437 | 437 | 437 | 437 |
| Samastipur | 20 | 380 | 380 | 380 | 380 | 380 |
| Begusarai | 18 | 342 | 342 | 342 | 338 | 342 |
| East champaran | 27 | 513 | 513 | 513 | 513 | 513 |
| West champaran | 18 | 342 | 342 | 342 | 342 | 342 |
| Saharsa | 10 | 190 | 190 | 190 | 190 | 190 |
| Khagaria | 7 | 133 | 133 | 133 | 133 | 133 |
| Gopalganj | 14 | 266 | 266 | 266 | 266 | 266 |
| Total | 137 | 2603 | 2603 | 2603 | 2599 | 2603 |
